# Adherence to a Traditional Mexican Diet Is Associated with Lower Hepatic Steatosis in US-Born Hispanics of Mexican Descent with Overweight or Obesity

**DOI:** 10.3390/nu15234997

**Published:** 2023-12-02

**Authors:** Melissa Lopez-Pentecost, Martha Tamez, Josiemer Mattei, Elizabeth T. Jacobs, Cynthia A. Thomson, David O. Garcia

**Affiliations:** 1 Sylvester Comprehensive Cancer Center, University of Miami Miller School of Medicine, Miami, FL 33136, USA; 2 Department of Nutrition, Harvard T.H. Chan School of Public Health, Harvard University, Boston, MA 02115, USA; m.tamez@mail.harvard.edu (M.T.); jmattei@hsph.harvard.edu (J.M.); 3 University of Arizona Cancer Center, The University of Arizona, Tucson, AZ 85724, USActhomson@arizona.edu (C.A.T.); davidogarcia@arizona.edu (D.O.G.); 4 Department of Epidemiology and Biostatistics, Mel and Enid Zuckerman College of Public Health, University of Arizona, Tucson, AZ 85724, USA; 5 Department of Health Promotion Sciences, Mel and Enid Zuckerman College of Public Health, University of Arizona, Tucson, AZ 85724, USA

**Keywords:** lifestyle, nutrition, liver disease, cancer risk, country of origin, health disparities, Latinos

## Abstract

Hispanics of Mexican descent have disproportionate rates of non-alcoholic fatty liver disease (NAFLD). The purpose of this work is to investigate the association between the traditional Mexican diet score (tMexS) and hepatic steatosis and fibrosis, two NAFLD-related clinical endpoints, in Hispanic adults of Mexican descent. Data from 280 Hispanic adults of Mexican descent (*n* = 102 men, 178 women) with overweight or obesity enrolled in a cross-sectional observational study were analyzed. The tMexS was calculated from 24 h dietary recalls. Hepatic steatosis and fibrosis measurements were assessed using transient elastography (Fibroscan^®^). Linear regression models testing the association between tMexS and hepatic steatosis and fibrosis were run individually and through the stratification of significant modifiers. Mean tMexS were 5.9 ± 2.1, hepatic steatosis scores were 288.9 ± 48.9 dB/m, and fibrosis scores were 5.6 ± 2.2 kPa. Among the US-born group, with every point increase in the tMexS, there was a statistically significant 5.7 lower hepatic steatosis point (95% CI: −10.9, −0.6, *p*-value = 0.07). Higher adherence to a traditional Mexican diet was associated with lower hepatic steatosis in US-born Hispanics of Mexican descent. Findings from the current work may serve to inform future culturally relevant interventions for NAFLD prevention and management in individuals of Mexican descent.

## 1. Introduction

Non-alcoholic fatty liver disease (NAFLD) is a known risk factor for hepatocellular carcinoma (HCC) [1,2], and its estimated prevalence among Hispanics is 63.7% [3]. These rates are well above those for other racial populations with rates for individuals of Mexican descent being particularly higher than for other Hispanic ethnic heritages [4,5]. NAFLD is on the trajectory to become the fastest growing risk factor for HCC in Hispanics residing in both the US and Mexico [6,7,8,9]. According to the American Association for the Study of Liver Diseases, modification of lifestyle behaviors, including the adoption of healthy eating patterns, is the cornerstone treatment for NAFLD [10,11]. However, there is a paucity of research evaluating the relationship between diet patterns and NAFLD risk or related risk factors and almost none in this high-risk group.

Dietary composition modulates the pathogenesis and trajectory of NAFLD [12,13]. A high intake of caloric-dense diets, particularly those rich in total fat and added sugars, has been implicated in the development and exacerbation of NAFLD [14]. Diets high in saturated and trans fats can contribute to lipid accumulation in the liver, leading to hepatic steatosis, a hallmark of NAFLD [13,15]. Furthermore, the excessive intake of sugars, especially fructose, has been associated with insulin resistance and lipogenesis in the liver [16,17]. In addition to macronutrients, oxidative stress of nutritional origin plays a crucial role in NAFLD [18,19]. The excessive intake of pro-oxidant nutrients, in combination with limited antioxidant defenses, leads to an imbalance and the generation of reactive oxygen species [18]. This oxidative stress contributes to liver inflammation and injury, further promoting the progression of NAFLD to more severe forms, such as non-alcoholic steatohepatitis (NASH) and fibrosis [18,19,20]. Among the behavior-related risk factors that place Hispanics of Mexican descent at greater risk for NAFLD are the high reported intakes of sugar-sweetened beverages and processed meat, which have been shown to increase the risk of developing this chronic condition [21,22,23,24]. However, there is limited research evaluating the relationship between eating patterns and NAFLD in this population despite the significantly elevated incidence of NAFLD and HCC burden. Studies among predominantly non-Hispanic White (NHW) populations support the premise that dietary patterns are a viable target for interventions leading to a reduction in NAFLD [25,26]. Previously, the Dietary Approach to Stop Hypertension (DASH) diet pattern was reported to significantly improve liver enzymes [26], a clinical measure suggestive of ongoing liver disease [6,27,28]. Likewise, robust literature supports the effectiveness of the Mediterranean diet pattern on improving hepatic steatosis and enzymes independent of weight loss [25,29]. Notably, in clinical practice, this dietary pattern has also been found to be the predominant diet recommended by primary care physicians to NAFLD patients [29]. However, potential barriers to consuming a Mediterranean-like diet pattern for Hispanics of Mexican descent have been documented [30], raising questions about the cultural relevance of recommending this diet for this population.

Studies examining more culturally relevant dietary patterns and the impact on any liver-related outcome in Hispanics of Mexican descent are sparse. One randomized, crossover feeding trial found that after 24 days a traditional Mexican dietary pattern intervention significantly improved insulin sensitivity among women of Mexican descent [31]. Epidemiological studies suggest that a higher adherence to a traditional Mexican diet is associated with favorable health outcomes including a reduced risk of obesity [32], obesity-related cancer mortality [33], and key metabolic components associated with NAFLD risk and cancer such as prediabetes [34], inflammation, and insulin resistance [31,35,36]. However, to our knowledge, no research to date has examined the association of this culturally relevant diet pattern and the pathological features of NAFLD in Hispanics of Mexican descent.

The purpose of this study was to examine the association between adherence to a traditional Mexican diet, assessed using the traditional Mexican diet score (tMexS), and hepatic steatosis and fibrosis in a sample of 280 Hispanic adults of Mexican descent with overweight and obesity. Further, we sought to evaluate the potential effect modification by multiple characteristics including sex [37], birthplace [6], and *PNPLA3* risk allele carrier status [38,39], factors previously identified to modify the relationship between diet and NAFLD and its risk factors. Findings from this work will contribute to the body of evidence guiding the development of future dietary interventions for NAFLD prevention and clinical management in this high-risk population.

## 2. Methods and Materials

### 2.1. Study Sample

This secondary analysis is an ancillary study to an Institutional Review Board (IRB)-approved observational cross-sectional study (IRB #1902380787; 11 May 2021) conducted among men and women of Mexican descent living in Southern Arizona. The details of the primary study have been previously summarized elsewhere [40]. Briefly, to be eligible for the primary study, individuals needed to self-identify as Mexican or from Mexican descent, be aged 18 to 64 years old, and have a body mass index (BMI) ≥25 kg/m^2^. Participants were excluded if they self-reported a previous diagnosis of liver disease or exceeded alcohol consumption of 21 standard drinks per week for men and 14 drinks per week for women [41]. Alcohol intake is a known independent risk factor for liver disease, and the inclusion of individuals in the study with excessive intake could introduce confounding variables, thereby confounding the nuanced interactions between other dietary factors and liver disease [42,43]. As part of the primary study, participants attended one study visit that took place in a clinic located in Tucson, Arizona, that specializes in the treatment of liver disease. Trained bilingual and bicultural study staff and graduate research assistants collected all study data. All study visits were conducted in the preferred language of each participant (English or Spanish) and, at the end of the study visit, participants were compensated USD 25.00 for their time.

### 2.2. Dietary Intake Assessment

Dietary intake was assessed through 24 h dietary recalls using the USDA multiple-pass method [44]. These were conducted on three non-sequential days of the week, two weekdays and one weekend day, during a period of two weeks after attending the clinical visit of the primary study. All participants in the current study sample completed all three dietary recalls and used a food amounts booklet provided to them to facilitate reporting of portions and amounts. Participants with an implausible energy intake below <500 and above >4000 were excluded from the analyses [35]. All 24 h recalls were administered by bilingual and bicultural trained personnel in the University of Arizona (UA) Cancer Center Behavioral Measurement and Interventions Shared Resource via telephone. Dietary data were processed using the Nutrition Data System for Research (NDSR-2019) [45]. Participants were compensated an additional USD 25.00 for completion of all three recalls, receiving a total of USD 50.00 if all study measures were completed.

### 2.3. Diet Pattern Scoring

The traditional Mexican diet pattern was previously identified from a historical review of food composition of traditional Mexican diets in Mexico and the US. It is composed of a mixture of Native Mesoamerican foods (pre-Hispanic) and Hispanic foods, characterized by high amounts of fruits, vegetables, complex carbohydrates, and corn-based dishes cooked with chilies, garlic, onions, herbs, beans, squash, citrus fruits, and rice [46]. From an extensive literature review, a traditional Mexican diet pattern has been converted into a composite score that can be calculated from food frequency data and 24 h dietary recalls [35,47]. The tMexS was based on methods by Santiago-Torres et al. [35] and adapted by Tamez et al. [47]. Briefly, the tMexS comprises 12 food group components (corn tortillas, beans and nuts, fruit, vegetables, rice, Mexican mixed dishes, poultry and eggs, fish, dairy products, red and processed meats, grains, and added sugars) that are categorized under traditional Mexican or US foods and altogether are computed to measure adherence to a traditional Mexican diet. Each component was created by adding the corresponding NDSR food subgroups using the average intake from the three dietary recalls. Each of the 12 components were scored using the sex-specific population median intake. A score of 0 was assigned to participants consuming below the median intake for traditional Mexican food components (corn tortillas, beans and nuts, fruit, vegetables, rice, Mexican mixed dishes, poultry and eggs, fish, and dairy products) or a score of 1 for participants consuming above the median intake. A reverse scoring was used for US food components (red and processed meats, refined grains other than rice, and added sugars). Possible scores for tMexS range from 0 to 12 with higher tMexS representing higher consumption of a more traditional Mexican diet pattern and less US foods. A summary of the scoring system is summarized in Supplementary Table S1.

### 2.4. Hepatic Steatosis and Fibrosis Assessment

Hepatic steatosis and fibrosis measurements were taken by a trained physician using transient elastography (TE) FibroScan^®^ (Echosens, Paris, France). This method is non-invasive, fast, reliable, and reproducible, with good intra- and interobserver levels of agreement [48] and has been validated against MRI, which is the imaging gold standard for liver disease [48,49]. TE measures hepatic fat infiltration as controlled attenuation parameter (CAP) scores and hepatic fibrosis expressed as kilopascals (kPa). Participants were asked to refrain from food and beverages, with the exception of water and black coffee or tea, for at least three hours before their assessment. A minimum interquartile range CAP score of 30 dB/m was established for each of the ten measurements taken per participant to establish score accuracy and quality assurance [50,51]. At the end of the primary study visit, each participant received a summary of their results and had the opportunity to review their results with a bilingual and bicultural physician or healthcare professional.

### 2.5. Covariate Assessment

As part of the primary study, participants were asked to complete questionnaires assessing key demographic, socioeconomic, sociocultural, health, behavior, and psychosocial characteristics. Data from these self-reported questionnaires were utilized as covariates to adjust the linear regression models. Anthropometric assessment was conducted by trained staff or graduate research assistant according to standard protocols [52]. Briefly, participant body weight was assessed with street clothing, without shoes, and on a calibrated scale to the nearest 0.1 kg (Tanita WB-100A). Height was assessed to the nearest 0.1 cm using a stadiometer. The measurements for body weight and height were utilized to calculate the participant’s BMI. Waist circumference was measured at the umbilicus using a Gulick measuring tape also recorded to the nearest 0.1 cm.

Two buccal swabs (Whatman, Maidstone, UK, OmniSwab) were collected from each participant to isolate genomic DNA to determine genotype status at the patatin-like phospholipase domain containing 3 (*PNPLA3*) gene (rs738409), given that this risk allele is highly prevalent in individuals of Mexican descent and associated with NAFLD risk and progression [53]. The UA Genetics Core isolated genomic DNA from buccal swabs, quantified it, and utilized it as a template in a TaqMan^®^ SNP Genotyping assay. This assay aimed to identify the genotype at the rs738409 SNP situated in codon 148 of *PNPLA3*. *PNPLA3* rs738409 genotype was used to define risk allele carrier status, with individuals categorized as CC, CG, and GG genotypes, corresponding to 0, 1, or 2 risk alleles.

First, unadjusted models were run, followed by models adjusted for literature-derived covariates (age, sex, BMI, energy intake, and leisure physical activity) (Model 1). Other relevant covariates were tested for substantial effect on model estimates (10% change). These included language spoken at home, income, birthplace, education, sedentary time, and *PNPLA3* risk allele (G) carrier status. Covariates with substantial effects on the model were language, birthplace, generation status, education, and *PNPLA3* risk allele carrier status. However, to prevent over-adjusting, a correlation analysis was conducted and the final covariates in Model 2 included covariates in Model 1 in addition to birthplace, education, and *PNPLA3* carrier status.

### 2.6. Statistical Analysis

Two-sample *t*-tests were used to test baseline differences in demographic and clinical characteristics across sex and birthplace and to calculate *p*-values for continuous variables including hepatic steatosis and fibrosis, age, and tMexS. For categorical variables, such as health insurance, education, language spoken at home, BMI category, *PNPLA3* risk allele carrier status, diabetes history, and cancer history, chi-squared tests were used. For variables that were non-normally distributed (BMI, energy intake, leisure activity, and sedentary time), Wilcoxon rank-sum tests were performed to test median differences across groups.

Main outcome variables, hepatic steatosis and fibrosis, were examined as continuous variables in linear regression analysis adjusted for covariates in different models. Effect modification was tested using an interaction term for sex, BMI, birthplace, language, education, income, and *PNPLA3* risk allele carrier status, individually. Linear regression models were then run, stratifying by factors that had a significant interaction. As an exploratory analysis, median consumption for each of the 12 tMexS components was examined and tested across groups using the Wilcoxon rank-sum test. All analyses were conducted using STATA/IC version 15.1 (StataCorp, College Station, TX, USA).

## 3. Results

### 3.1. Sample Characteristics

A total of 280 Hispanic adults of Mexican descent in Southern Arizona with overweight or obesity were included in this secondary analysis. There were a total of 102 men and 178 women with a mean overall age of 44.8 ± 11.1 years old. Mean hepatic steatosis was 288.9 ± 48.9 dB/m, for which a CAP score of 288 dB/m is equivalent to 5% hepatic steatosis and indicative of NAFLD [51]. Mean hepatic fibrosis was 5.6 ± 2.2 kPa. Participant characteristics by sex and birthplace are summarized in Table 1. When comparing participant characteristics by sex, women were statistically significantly older (*p*-value = 0.01) with a higher BMI (*p*-value = 0.04) and reported consuming significantly less calories (*p*-value < 0.001) compared to men. When further comparing participants by birthplace, both men and women born in Mexico compared to their US-born counterparts had a lower proportion of individuals with health insurance (men: *p*-value = 0.03, women: *p*-value < 0.001), and a higher percentage spoke Spanish at home (men: *p*-value < 0.001, women: *p*-value < 0.001), had a lower mean BMI (men: *p*-value = 0.04, women: *p*-value = 0.003), and had higher tMexS (men: *p*-value = 0.01, women: *p*-value < 0.001). Additionally, US-born men were statistically significantly older then Mexican-born men (*p*-value < 0.001) and US-born women self-reported higher formal education (*p*-value < 0.001) and higher sedentary time compared to Mexican-born women (*p*-value < 0.001).

### 3.2. Diet Scores and Liver Steatosis and Fibrosis

Mean tMexS in the study sample were 5.9 ± 2.1. Individual means by sex and birthplace are summarized in Table 1. Overall, US-born men had the lowest tMexS (5.0 ± 2.1), and Mexico-born women had highest scores (6.3 ± 2.0).

Linear regression analyses between tMexS and hepatic steatosis and fibrosis showed no significant associations for any of the models (Table 2). The results showed an inverse association between tMexS and steatosis across all models, though these were not statistically significant.

Effect modifications between tMexS and hepatic steatosis and fibrosis by sex, BMI, birthplace, language spoken at home, education, income, and *PNPLA3* risk allele carrier status were examined. The results showed that sex modified the relationship between tMexS and hepatic steatosis (P-interaction = 0.04), and fibrosis (P-interaction = 0.04). Birthplace (P-interaction = 0.02) and *PNPLA3* risk allele carrier status (P-interaction = 0.04) modified the relationship between tMexS and hepatic steatosis but not fibrosis.

Analyses stratified by birthplace showed a statistically significant relationship between tMexS and hepatic steatosis only among the US-born group (Table 3). With every point increase in tMexS, there was a concurrent 5.7-point decrease in hepatic steatosis scores (95% CI: −10.9, −0.6, *p*-value = 0.03). No other significant findings were observed among the subgroups for liver steatosis or fibrosis.

**Figure 1:**
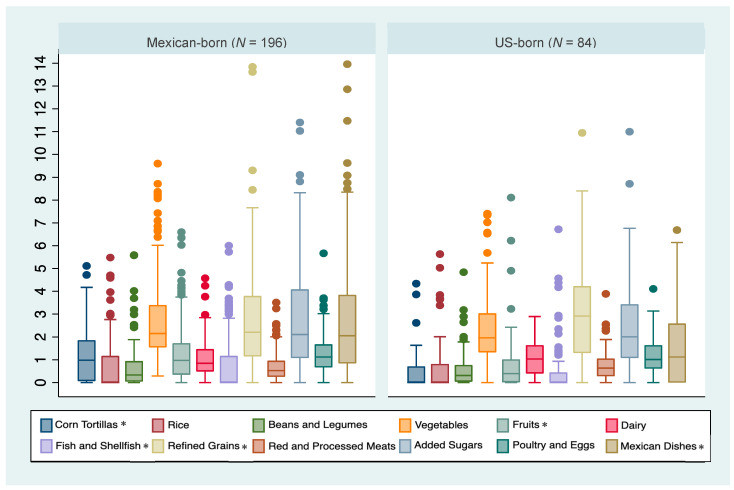
Distribution of servings for each Traditional Mexican Diet (tMexS) score category by birthplace in a sample of Hispanic adults of Mexican descent in Southern Arizona (*n* = 280). * Indicates statistically significant differences across birthplace groups at *p*-value < 0.05.

Median intakes and interquartile ranges of each tMexS component by birthplace are summarized in Figure 1. Compared to the US-born group, the Mexico-born group had higher median intakes of corn tortilla (*p*-value < 0.001), whole fruits (*p*-value < 0.001), fish and shellfish (*p*-value = 0.01), and Mexican mixed dishes (*p*-value = 0.002) and a lower median consumption of refined grains (other than rice) (*p*-value = 0.02). No other statistically significant differences in median intake values were observed.

## 4. Discussion

The current study investigated the relationship between a culturally relevant diet score, the tMexS, which measures adherence to a traditional Mexican diet, and hepatic steatosis and fibrosis in a sample of Hispanic adults of Mexican descent with overweight or obesity living in Southern Arizona. The results demonstrate a non-significant inverse association between the tMexS and hepatic steatosis in the total study sample. When investigating the potential effect modification, sex modified the association between tMexS and hepatic steatosis and fibrosis, while birthplace and *PNPLA3* risk allele carrier status modified the relationship between tMexS and hepatic steatosis only. Stratified analyses showed a statistically significant relationship between tMexS and hepatic steatosis only for US-born men. To our knowledge, this is the first study to investigate the relationship between a diet pattern score and hepatic steatosis and fibrosis, two clinical endpoints of NAFLD, in a sample of Hispanic adults of Mexican descent, a population consistently shown to have some of the highest rates of this disease.

The mean tMexS in the current analysis are comparable to those previously reported by Tamez et al. [47] and Santiago-Torres and colleagues [35] with mean scores in the current study being slightly higher. In the work by Tamez and colleagues including data from 3542 Mexican heritage individuals from the Hispanic Community Health Study/Study of Latinos, the mean traditional Mexican diet score was 5.8 ± 0.05. In the work by Santiago-Torres and colleagues, which utilized data from 493 postmenopausal women of Mexican decent, the mean traditional Mexican diet scores were 5.8 ± 2.1. In the current study, mean scores were 5.9 ± 2.1 in the overall sample and 6.3 ± 2.0 among Mexican-born women. Scores in our study, particularly those for women, may be slightly higher than those in prior work due to the geographical location of the study participants, which is in close proximity to Mexico and in a highly dense Hispanic–Mexican community. Regarding hepatic steatosis and fibrosis, the results are comparable to those reported in a study of the Cameron County Hispanic Cohort by Watt and colleagues [54]. In their study of Mexican American Hispanics, men and women had a median hepatic steatosis score of 306 dB/m and 277 dB/m, respectively, comparable to the men (291.0 dB/m) and women (287.6 dB/m) in the current study. It should be noted that Watt et al. [55] presented medians versus means; therefore, direct comparisons may be hindered.

The literature on diet and NAFLD for Hispanic individuals is sparse and further limited for Hispanics of Mexican descent, making the direct comparison of results challenging. The majority of studies have evaluated dietary patterns like the DASH pattern [56,57,58], Mediterranean pattern [25,57,59,60,61], or variations of the Healthy Eating Index [56,59], in predominantly NHW populations [25,59,60,61,62], with few among diverse populations [56,57,58]. In these studies, NAFLD-related outcomes have varied with outcomes including liver fat [25,59], liver damage [60], liver enzymes [26,60], and odds or the risk for NAFLD [56,58,61,62]. Consistent with previous research [37,63], the mean hepatic steatosis was higher among men compared to women. There was a stronger relationship between tMexS and hepatic steatosis among men, although these results were not statistically significant, possibly due to sample size. Birthplace significantly modified the relationship between tMexS and hepatic steatosis. Consistently, the literature highlights differences in the prevalence and presence of NAFLD risk factors for individuals born in Mexico versus the US [6,64,65,66]. One study by Flores and colleagues compared the prevalence of chronic liver disease risk factors in a sample of Mexican individuals born and residing in Mexico vs. the US [6]. Findings from this work indicate that US-born Mexicans have a 1.4 (1.1–1.9) higher odds of having metabolic syndrome, 3.0 (1.9–4.8) higher odds of having diabetes, 3.9 (3.1–4.9) higher odds for obesity, and 5.4 (2.9–10.1) higher odds of having abdominal obesity, compared to Mexico-born individuals. Similar to the current study, US-born individuals had statistically significant higher BMIs compared to their Mexico-born counterparts. Interestingly, our results indicate that US-born individuals of Mexican descent may benefit more from following a more traditional Mexican diet than their Mexican-born counterparts. A potential explanation for this may be that the adoption of healthier diet habits may lead to better health outcomes, especially for individuals who start off with poorer eating habits [67]. For example, a study by Grafenauer et al. demonstrated that individuals who started off with unhealthier diet habits benefitted more from weight loss interventions as compared to those who started with healthier food choices [68]. Similarly, in the current study, it may be that adopting a traditional Mexican diet pattern may be particularly beneficial for US-born individuals who may have unhealthy dietary habits reflective of higher acculturation as compared to healthier dietary habits for less acculturated, Mexico-born individuals. However, more research is needed to better understand how and why this may occur.

In the current analysis, a significant interaction was found for PNPLA3 rs738409 risk allele carrier status in that it modified the relationship between tMexS and hepatic steatosis, although no significant relationship was observed once stratification analyses were conducted. The rs738409 (C > G) single-nucleotide polymorphism has been shown to increase the susceptibility and severity of NAFLD [53]. Our results are consistent with those in the literature who have found that *PNPLA3* risk allele carrier status modified the relationship between certain dietary factors and hepatic steatosis [38,69]. Although precision nutrition in the clinical setting remains a theoretical practice, reports indicate approximately 75,000 genetic tests currently on the market with around 10 new ones entering the market daily [70]. The current findings emphasize the viability of integrating a culturally pertinent dietary paradigm as a prospective approach to reduce the incidence of NAFLD and HCC among US-born Hispanic individuals. Additionally, these results may catalyze the development of culturally targeted lifestyle interventions and dietary recommendations, potentially heightening the overall interest and engagement of individuals in seeking consultations with dietitians.

While national dietary guidelines for the treatment of NAFLD do not currently exist, the EASL-EASD-EASO Clinical Practice Guidelines have recommended the Mediterranean diet as the diet of choice for treatment of NAFLD [29]. The Mediterranean diet has been found to be among the most effective diets to improve cardio-metabolic factors associated with NAFLD and to induce weight loss [71], the most effective way to reduce hepatic steatosis and improve histopathological features of NASH [11]. However, barriers to engage in such dietary modifications and long-term weight loss maintenance have been difficult among Hispanic patients for a variety of reasons [30,72]. First, the cultural relevance and overall acceptability of the Mediterranean diet among Hispanics may be questionable. A study by Estrada Del Campo and colleagues reported that Hispanic American women who engaged in a Mediterranean diet intervention aimed at reducing cardiometabolic factors reported that they or their families disliked the suggested foods or that the cost of the suggested foods was too high [30]. A lack of cultural relevance of lifestyle programs and dietary recommendations may also have an impact on the overall interest of patients in procuring dietician consults. A study by Saeed et al. reported that provider referral to dietetic services for the management of NAFLD was hindered by several factors, including patients’ overall low interest in acquiring such services and the availability of dieticians and lifestyle programs [73]. In this regard, a traditional Mexican diet poses a promising dietary strategy for NAFLD prevention and management while sustaining traditional and familiar dietary choices and thus addressing acceptability concerns observed for other dietary recommendations such as with the Mediterranean diet.

A traditional Mexican diet holds promise in the prevention and management of NAFLD through the modulation of various molecular pathways. One potential mechanism of action could be through the diet’s high fiber content, derived from fruits, vegetables, and whole grains, which may influence gut microbiota composition and function, thereby impacting short-chain fatty acid production [74]. These fatty acids, in turn, have been linked to anti-inflammatory and lipid-metabolism-modulating effects [75], potentially mitigating hepatic lipid accumulation and the inflammation characteristic of liver disease [76]. Furthermore, traditional Mexican foods contain bioactive compounds, such as polyphenols and flavonoids, which may exert antioxidant properties, counteracting oxidative stress implicated in NAFLD pathogenesis [20,77]. The diet’s well-balanced macronutrient profile of healthy fats from sources like avocados and omega-3 fatty acids from fish might contribute to lipid homeostasis that benefits hepatic steatosis [78]. The potential interplay of these molecular pathways underscores the scientific rationale supporting further research efforts to more granularly understand the preventive effects of a traditional Mexican diet on NAFLD.

### Strengths and Limitations

Several strengths exist in the current study. First, this analysis addresses a significant gap in the literature given that the evidence on diet and NAFLD-related outcomes among Hispanics of Mexican descent is scarce. Second, we expand on the existing literature by including a culturally relevant dietary score for a population underrepresented in NAFLD research [79] despite their disproportionate burden of this disease. This work represents a steppingstone for building the necessary evidence for the implementation of culturally relevant dietary guidelines to address the NAFLD disparities observed for this group. However, future research should evaluate the effectiveness and clinical utility of current NAFLD management guidelines for this at-risk population to ensure that efforts to manage this condition are equitable and accessible to those who need them most. Lastly, the study sample in the current study was well characterized and allowed for the appropriate evaluation of covariates. While the strengths of this study are evident, limitations of the current work also exist. First, while dietary studies are crucial for understanding the impact of nutrition on various health outcomes, these face significant limitations due to the inherent challenges in accurately measuring individuals’ dietary exposures. The self-reported dietary intake assessment with 24 h dietary recalls is subject to recall and reporting biases, which may have affected the accuracy of our data. Additionally, the inclusion of only individuals with overweight or obesity hinders our ability to account for a wider range of exposures and interactions in the current analysis including the generalizability to “lean” NAFLD patients [80]. Future research should comprise a larger and more clinically diverse sample that includes normal-weight participants with a variety of body compositions to allow for adequately powered sub-analyses. For example, this would allow an evaluation of the relationship between a traditional Mexican diet and hepatic steatosis among US-born individuals that are not carriers of the *PNPLA3* risk allele.

## 5. Conclusions

Results of this study indicate that the interplay of birthplace and the presence of the PNPLA3 risk allele in overweight or obese Hispanic adults of Mexican descent could influence the association between the consumption of a traditional Mexican diet and hepatic steatosis. This work contributes to the sparse body of literature exploring the relationship between dietary patterns and NAFLD outcomes in Hispanic adults of Mexican descent. Findings from the current study seek to guide the development of forthcoming culturally targeted interventions aimed at preventing and managing NAFLD within this specific population.

## Figures and Tables

**Table 1:** Baseline characteristics of Hispanic adults of Mexican descent in Southern Arizona (*N* = 280) by sex and birthplace.

	Men			Women		
	Total (*n* = 102, 36%)	Mexico-Born (*n* = 70, 68%)	US-Born (*n* = 32, 32%)	Total (*n* = 178, 64%)	Mexico-Born (*n* = 126, 71%)	US-Born (*n* = 52, 29%)
Liver steatosis (CAP dB/m, mean ± SD)	291.0 ± 49.7	293.1 ± 49.6	286.6 ± 50.6	287.6 ± 48.5	290.6 ± 48.5	280.5 ± 48.1
Liver fibrosis (kPa, mean ± SD)	5.8 ± 2.4	5.8 ± 2.6	5.7 ± 1.7	5.5 ± 2.1	5.5 ± 2.1	5.4 ± 1.9
Age (years, mean ± SD) ^ab^	42.6 ± 12.1	45.9 ± 10.8	35.5 ± 11.9	46.0 ± 10.3	46.6 ± 9.7	44.5 ± 11.7
Health insurance (*n*, %) ^bc^						
Yes	67 (65.7)	41 (58.6)	26 (81.3)	104 (58.4)	56 (44.4)	48 (92.3)
Education (*n*, %) ^c^						
Less than high school	25 (24.5)	22 (31.4)	3 (9.4)	55 (30.9)	45 (35.7)	10 (19.2)
High school or GED	23 (22.6)	15 (21.4)	8 (25.0)	39 (21.9)	33 (26.2)	6 (11.5)
Greater than high school	54 (52.9)	33 (47.1)	21 (65.6)	84 (47.2)	48 (38.1)	36 (69.2)
Language in the home (*n*, %) ^bc^						
English	27 (26.2)	4 (5.7)	23 (71.9)	49 (27.5)	12 (9.5)	37 (71.2)
Spanish	75 (73.6)	66 (94.3)	9 (28.1)	129 (72.4)	114 (90.5)	16 (30.8)
BMI (kg/m^2^, median, IQR) ^abc^	30.6 (25.4, 45.1)	30.1 (25.4, 44.7)	32.5 (25.9, 45.1)	32.2 (25.0, 55.5)	31.4 (25.0, 55.5)	34.1 (25.0, 51.1)
BMI Classification (*n*, %) ^c^						
Overweight (25–29.9 kg/m^2^)	44 (43.1)	34 (48.6)	10 (31.2)	57 (32.0)	47 (37.3)	10 (19.2)
Obesity (≥30 kg/m^2^)	58 (56.9)	36 (51.4)	22 (68.8)	121 (68.0)	79 (62.7)	42 (80.8)
*PNPLA3* risk allele status (*n*, %)						
No risk allele (CC)	22 (21.6)	14 (20.0)	8 (25.0)	46 (25.8)	35 (27.8)	11 (21.2)
At least one risk allele (CG or GG)	80 (78.4)	56 (80.0)	24 (75.0)	132 (74.2)	91 (72.2)	41 (78.8)
Diabetes (*n*, %)	9 (8.8)	4 (5.7)	5 (15.6)	18 (10.1)	12 (9.5)	6 (11.5)
Total energy intake, kcals/day ^a^ (median, IQR)	1679.3 (729.9, 3947.5)	1684.9 (729.9, 3947.5)	1642.9 (1007.2, 3013.9)	1299.2 (500.6, 2980.4)	1296.2 (519.9, 2980.4)	1309.4 (500.6, 2500.5)
Leisure activity, min/week (Median, IQR)	120 (0, 1680)	110 (0, 960)	120 (0, 1680)	90 (0, 960)	90 (0, 960)	80 (0, 420)
Sedentary time, hours/week ^c^ (Median, IQR)	5.0 (1.0, 14.0)	4.8 (1.0, 14.0)	6.5 (1.5, 14)	5.0 (0.33, 20.0)	4.0 (0.33, 14.0)	8.8 (1.0, 20.0)
Traditional Mexican diet score (tMexS) (mean ± SD) ^abc^	5.8 ± 2.1	6.2 ± 1.9	5.0 ± 2.1	6.0 ± 2.1	6.3 ± 2.0	5.3 ± 2.0

^a^ Statistically significant differences between all men and all women. ^b^ Statistically significance differences between Mexico and US-born men. ^c^ Statistically significance differences between Mexico and US-born women. Statistical significance at a *p* value ≤0.05. *p*-value calculation: *t* test (continuous) and chi-squared test (categorical), Wilcoxon Rank Sum (non-parametric).

**Table 2:** Linear regression analysis between the traditional Mexican diet Score (tMexS) and hepatic steatosis (CAP, dB/m) and fibrosis (kPa) in a sample of Hispanic adults of Mexican descent in Southern Arizona (*N* = 280).

	Crude Model			Model 1 ^a^			Model 2 ^b^		
	Estimate	95% CI	*p*-Value	Estimate	95% CI	*p*-Value	Estimate	95% CI	*p*-Value
Steatosis									
tMexS	−1.8	−4.6, 0.9	0.20	−0.06	−2.7, 2.6	0.96	−0.8	−3.4, 1.9	0.57
Fibrosis									
tMexS	−0.06	−0.2, 0.07	0.37	0.02	−0.1, 0.1	0.68	0.02	−0.1, 0.1	0.81

^a^ Model 1 adjusted for age, sex, BMI, energy intake, and leisure physical activity. ^b^ Model 2 adjusted for age, sex, BMI, energy intake, leisure physical activity, language, birthplace, education, and PNPLA3.

**Table 3:** Relationship between the Traditional Mexican diet Score (tMexS) and hepatic steatosis score (CAP, dB/m) and fibrosis (kPa) stratified by sex, birthplace, and genetic risk allele status in a sample of Hispanic adults of Mexican descent in Southern Arizona (*N* = 280).

	Hepatic Steatosis			Hepatic Fibrosis		
	Estimate	95% CI	*p*-Value	Estimate	95% CI	*p*-Value
**Sex**						
Male (*n* = 102)	−3.3	−7.6, 1.1	0.14	0.22	−0.01, 0.5	0.07
Female (*n* = 178)	0.90	−2.5, 4.3	0.60	−0.11	−0.3, 0.04	0.14
**Birthplace**						
Mexico-born (*n* = 196)	1.5	−1.7, 4.7	0.35	0.05	−0.1, 0.2	0.51
US-born (*n* = 84)	−5.7	−10.9, −0.6	0.03	−0.09	−0.3, 0.1	0.41
**Risk allele status**						
No risk allele (**CC**) (*n* = 68)	−6.1	−12.7, −0.4	0.07	−0.04	−0.27, 0.2	0.74
One or more risk allele (**CG** or **GG**) (*n* = 212)	1.3	−1.6, 4.2	0.38	0.05	−0.1, 0.2	0.52

All models adjusted for age, sex, BMI, energy intake, leisure physical activity, language, birthplace, education, and *PNPLA3*.

## Data Availability

The data presented in this study are available on request from the corresponding author. The data are not publicly available due to privacy or ethical restrictions.

## Supplementary Materials

The following supporting information can be downloaded at https://www.mdpi.com/article/10.3390/nu15234997/s1: Supplementary Table S1. Description of the traditional Mexican diet score (tMexS).
