# Supplementary material for: Adherence to a Traditional Mexican Diet Is Associated with Lower Hepatic Steatosis in US-Born Hispanics of Mexican Descent with Overweight or Obesity
Source: Nutrients. 2023 Dec 2;15(23):4997. doi: 10.3390/nu15234997 (PMC10708445; doi:10.3390/nu15234997)
Supplement: Supplementary file 1 [file nutrients-15-04997-s001.zip › nutrients-2707862-supplementary.pdf]

Supplementary Table S1. Description of the traditional Mexican diet score (TMexS)

Scoring Components	Men Median (svg/day)	Women Median (svg/day)	Point
**Traditional Mexican foods**			
1. Whole fruit	0.58	0.84	> Median + 1
2. Vegetables	2.00	2.27	
3. Beans and legumes	0.44	0.29	
4. Corn tortilla	0.81	0.56	
5. Rice	0.00	0.00	
6. Dairy	0.90	0.96	
7.Mexican Mixed dishes + Soups	1.80	1.75	
8. Fish and shellfish	0.00	0.00	
9. Poultry and eggs	1.50	0.93	
**US / American Foods**			
10. Processed and red meats	0.77	0.48	< Median + 1
11. Added sugars	2.56	1.97	
12. Refined grains	3.18	3.18	
		**Score Range**	0-12 points
